# Evaluation of miRNA Expression in Pediatric Cirrhosis Caused by BA and PFIC

**DOI:** 10.1155/bmri/4087823

**Published:** 2026-01-31

**Authors:** Fatemeh Khosravi, Ramin Yaghobi, Afsoon Afshari, Nasrin Motazedian, Bita Geramizadeh, Tayebeh Kazemi, Seyed Mohsen Dehghani

**Affiliations:** ^1^ Transplant Research Center, Shiraz University of Medical Sciences, Shiraz, Fars Province, Iran, sums.ac.ir; ^2^ Nephro-Urology Research Center, Shiraz University of Medical Sciences, Shiraz, Fars Province, Iran, sums.ac.ir; ^3^ Otolaryngology Research Center, Shiraz University of Medical Sciences, Shiraz, Fars Province, Iran, sums.ac.ir

**Keywords:** biliary atresia, cholestasis, liver cirrhosis, microRNA, pediatric, progressive familial intrahepatic cholestasis

## Abstract

Liver cirrhosis is one of the most common causes of death for pediatric patients with cholestasis. Because microRNA (miRNA) plays a part in the pathogenesis of the disease, comparing the changes in miRNA expression in cholestatic patients with liver cirrhosis could be helpful for therapeutic or prognostic purposes. miRNA levels in cirrhotic pediatric patients with progressive familial intrahepatic cholestasis (PFIC) and biliary atresia (BA) were studied in this research to comprehend the molecular distinctions and the significance of miRNA regulation in fibrosis and liver cirrhosis. Blood samples were collected from 43 PFIC patients and 84 BA patients, all of whom were confirmed to have liver cirrhosis. The in‐house SYBR Green RT‐qPCR techniques were established to assess the variations in the expressions of 12 miRNAs. Utilizing bioinformatics tools, the gene targets of the studied miRNAs were examined. Patients′ expression of 12 miRNAs was higher than that of the healthy group. However, there was a notable distinction between the miRNAs in PFIC and BA. The levels of miR‐34, miR‐155, miR‐199, miR‐200b, and miR‐222 were significantly higher in BA than in PFIC. PFIC showed a significantly higher level of miR‐223 than BA. The predictive importance of distinct miRNAs in both diseases was revealed by the AUC values. In BA, miR‐222 was associated with both liver transplantation and the PELD score. Death was correlated with miR‐21, miR‐155, miR‐199, and miR‐200 in BA and miR‐34 in PFIC. The analysis showed the significance of the connection between miRNA expression and PI3K/Akt and TGF‐*β* signaling in pediatric liver cirrhosis. Future investigations can evaluate the significance of the studied miRNAs as novel therapeutic targets and diagnostic options.

## 1. Introduction

Liver cirrhosis is the ultimate result of cholestasis, a category of liver illnesses that is initially characterized by jaundice and cholestasis. Cholestasis can be classified as either biliary atresia (BA) or non‐BA, including progressive familial intrahepatic cholestasis (PFIC) [1, 2]. BA, the most prevalent liver condition in infancy, is a major contributor to obstructive jaundice and can result in a high newborn mortality rate [3]. BA has a 2‐year lethal potential if left untreated. The disease begins with a destructive inflammation of the liver, which progresses to fibrosis and cirrhosis. Because of growing liver fibrosis, Kasai portoenterostomy (KPE), the most significant treatment for BA, seldom succeeds, and most patients eventually need a liver transplant [4]. PFIC is caused by autosomal recessive mutations that interfere with the function of liver cells in forming and excreting bile [5]. Cholestasis and subsequent outcomes are the usual symptoms of the condition throughout the first year of life. In the first few months after birth, it frequently presents as severe pruritus, slowed weight gain, malnourishment, cholestatic jaundice, and hypocholesterolemia [6]. Both BA and PFIC cholestatic disorders typically develop into fibrosis and cirrhosis prior to adulthood. The management of cholestatic disorders in pediatric patients with cirrhosis has proven difficult because of inadequate treatment, delayed diagnosis, and a lack of knowledge about the exact mechanisms underlying the condition [7]. Children with liver cirrhosis have a higher risk of death. Studies on pediatric cirrhosis have been lacking, and further study is required to have a better understanding of the condition, particularly to identify therapeutic options and enable prompt and simple diagnosis. Recently, gene therapy, specifically microRNAs (miRNAs), has shown promise as potential targets for the development of novel liver disease treatment approaches [8]. miRNAs are important for the pathophysiology of liver injury, as evidenced by the significant attention given to changes in miRNA expression that take place during the beginning and/or progression of liver disorders [9]. On the other hand, because of their complicated chemical structure, great stability, and lack of postprocessing alterations, miRNAs have been the subject of numerous investigations into their diagnostic and prognostic potential as useful biomarkers in liver illnesses [10]. Consequently, it can be informative to compare the changes in miRNA expression in the cirrhotic stage of cholestasis patients with various etiologies. Comparing these variations in expression can reveal the significance of each miRNA in these conditions, but it can also reveal novel therapeutic approaches for patients with liver cirrhosis. The objective of the present study was to evaluate the significance of miRNAs in the fibrogenesis of liver cirrhosis in pediatric patients with BA and PFIC.

## 2. Materials and Methods

### 2.1. Patients and Healthy Group Samples

**Figure 1:**
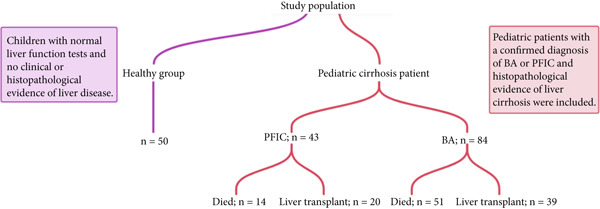
Flow diagram for the process of selection of patients. The study included 177 patients in total: 50 healthy groups, 43 PFIC patients, and 83 BA patients. The number of liver transplant recipients and the number of deaths in each category are displayed in the flowchart. SYBR Green Real‐time PCR was used to evaluate the expression of miRNAs in samples from these patients.

The samples gathered were a part of the Shiraz Pediatric Liver Cirrhosis Cohort Study (SPLCCS) and were collected between 2018 and 2023 (IR.SUMS.REC.1398.142) [11]. For this investigation, 84 pediatric patients with BA and 43 pediatric patients with PFIC were chosen based on their clinical and pathological results supporting the diagnosis of liver cirrhosis, which was validated by a gastroenterologist. Excluded from the study were additional pediatric samples that had pathological and clinical findings other than BA and PFIC (Figure 1). The pediatric patients′ liver cirrhosis registry was utilized to obtain their laboratory and clinical information (IR.SUMS.REC.1399.530). The serum and plasma separated from the blood samples taken were kept in a freezer at −80°C. As a healthy group, blood samples from 50 healthy group were also gathered. The samples were collected in accordance with the ethics code (IR.SUMS.REC.1402.032). All participants in this study provided informed consent from their parents or legal guardians before blood samples were collected.

Using biochemical tests, liver factors in serum samples from pediatric patients with BA and the healthy group were assessed [12]. Ten tests, including ALT, AST, creatinine, ALK, total bilirubin, albumin, direct bilirubin, BUN globulin, and total protein, were performed and compared with the cohort samples along with the demographic information of these children (Table 1).

**Table 1:** Properties of children, demographic and descriptive characteristics in study groups.

**Parameters**	**Healthy group**	**BA**	**PFIC**	**p** **value**
Gender				
Female	52.0 (26)	46.4 (39)	55.8 (24)	0.94
Male	48.0 (24)	53.6 (45)	44.2 (19)	
Age	6.02 ± 1.55	5.26 ± 3.08	5.98 ± 2.95	0.19
Blood chemistry test (mean ± SD)				
Creatinine (mg/dL)	0.51 ± 0.086	0.44 ± 0.21	0.44 ± 0.21	0.001
AST	18.08 ± 6.86	270.50 ± 261.37	389.41 ± 699.44	0.001
ALT	6.14 ± 3.28	173.53 ± 149.93	205.83 ± 315.44	0.001
ALK	511.48 ± 156.8	1336.13 ± 797.83	1093.43 ± 1117.24	0.001
Total bili	0.41 ± 0.10	13.78 ± 9.98	13.47 ± 11.05	0.001
Direct bilirubin	0.10 ± 0.05	9.18 ± 8.33	8.76 ± 8.01	0.001
Albumin	4.72 ± 0.37	3.39 ± 0.58	3.80 ± 0.73	0.001
BUN	10.89 ± 2.73	13.47 ± 11.05	13.47 ± 11.05	0.89
Globulin	2.46 ± 0.39	3.19 ± 0.91	3.00 ± 1.03	0.001
Total protein	7.04 ± 1.16	6.36 ± 1.06	6.72 ± 0.96	0.001

### 2.2. Total RNA Isolation and cDNA Synthesis

Blood cells were isolated using Ficoll from the study participants′ blood samples; before being used for RNA extraction, they were washed with phosphate buffer. A −80°C freezer was used to preserve the separated cell samples. For this study, total RNA was isolated using Trizol (Invitrogen, Carlsbad, CA, United States). Using Nanodrop (Thermo Fisher Scientific, United States) with absorbance ratios of 260/280 nm, we assessed the amount and quality of total RNA extracted from the samples. To synthesize cDNA, we employed a stem–loop primer in a reverse transcriptase reaction. This reaction was carried out using a UROX kit, and each sample′s template was 1 ng of total RNA [13].

### 2.3. Quantitative RT‐PCR Detection for miRNA

Using RT‐qPCR, the expression of 12 miRNAs and U6, an internal healthy group gene, was assessed. Table 2 lists the primers created for each of these miRNAs. A total of 10 *μ*L of mix, 2 *μ*L of cDNA as template, 1 *μ*L of primers (5 pm) per reaction, and up to 20 *μ*L of DEPC water were used in the SYBR Green reaction. For a total of 40 cycles, the StepOne ABI Applied Biosystems (Life Technologies) was used at 95°C for 15 min, 95°C for 15 s, and 62°C for 60 s. All miRNA expression levels were investigated using Livak′s method (*ΔΔ*Ct^−2^) to calculate the average cycle threshold (Ct) of the measured values.

**Table 2:** miRNA primer sequences and RT‐qPCR parameters.

**miRNA**	**Sequence (5** ^′^ **to 3** ^′^ **)**	**Length**	**RT-PCR condition**	**Reaction mixture**
U6	CTCGCTTCGGCAGCACA	17	1 cycle: 95°C/5 min; 40 cycles: 95°C/15 s, 62°C/35 s, 62°C/1 min followed by melt curve	10 *μ*L of Master mix, 0.2 *μ*L of ROX solution, 1 *μ*L of primer (5 pm), and 2 *μ*L of cDNA template, Add water up to a total volume of 20 *μ*L.
has‐miR‐200b‐3p	TAATACTGCCTGGTAATGATGA	22		
hsa‐miR‐155‐5p	TTAATGCTAATCGTGATAGG	20		
has‐miR‐21‐5p	TAGCTTATCAGACTGATGTTGA	22		
has‐miR‐29a‐3p	TAGCACCATCTGAAATCGGTTA	22		
hsa‐miR‐199a‐5p	CCCAGTGTTCAGACTACCTGTTC	23		
hsa‐miR‐33a‐3p	CAATGTTTCCACAGTGCATCAC	22		
hsa‐miR‐34a‐5p	TGGCAGTGTCTTAGCTGGTTGT	22		
hsa‐miR‐122‐5p	TGGAGTGTGACAATGGTGTTTG	22		
hsa‐miR‐192‐5p	CTGACCTATGAATTGACAGCC	21		
hsa‐miR‐214‐5p	TGCCTGTCTACACTTGCTGTGC	22		
hsa‐miR‐222‐3p	AGCTACATCTGGCTACTGGGT	21		
hsa‐miR‐223‐3p	TGTCAGTTTGTCAAATACCCCA	22		

### 2.4. Data Analysis

SPSS software Version 23 was used to analyze the research results, and GraphPad Prism software was used to create the graphics. In order to compare the features assessed in the healthy group and patient groups, the *t*‐test, Mann–Whitney statistical tests, and one‐way ANOVA were employed. Using Livak′s method, we conducted RT‐qPCR data analysis. Using GraphPad Prism, the ROC curves and the area under the curve (AUC) were computed and illustrated. Using Spearman and Pearson correlation, we assessed the relationship between miRNAs and patient characteristics [14]. After the data were analyzed, heatmap graphics were created using R software. One of the packages utilized was heatmap. Data were entered into the software as a matrix in order to execute hierarchical clustering based on the similarity between samples and features. The heatmaps were used to visually represent correlation patterns.

### 2.5. Analysis of Molecular Interaction Network

In this study, the target genes of miRNAs in liver fibrosis, BA, and PFIC were initially identified by extracting the genes mentioned in scientific literature about the disease that are thought to be possible miRNA target genes. The molecular mechanisms linked to liver cirrhosis are mostly influenced by these genes. Gene networks were analyzed and modeled using Cytoscape software after these genes were identified. An illustration of the relationships between the revealed genes and target miRNAs was provided in these genomic maps. In this network, every node represents a gene or miRNA, and the edges, or links, show how they might interact. By examining the intricate connections between genes and target miRNAs in greater detail, this analysis method will allow us to better understand how these linkages are regulated and how they affect the pathophysiology of cirrhosis.

## 3. Results

### 3.1. Baseline Characteristics and Clinical Outcomes

Demographic data and liver‐related biochemical tests were statistically analyzed for 177 participants in this study, which included a healthy group (*n* = 50) and pediatric cirrhotic patients (*n* = 127) (Figure 1). The mean ages of the PFIC group, the BA group, and the healthy group were 5.98 ± 2.95, 5.26 ± 3.08, and 6.02 ± 1.55 years, respectively. In every liver function test, BA and PFIC patients differed significantly from the healthy group. In comparison to the healthy group, pediatric patients with BA and PFIC exhibited statistically significant (*p* value < 0.05) increases in mean levels of aspartate aminotransferase, alanine aminotransferase, albumin, globulin, creatinine, total protein, alkaline phosphatase, total bilirubin, and direct bilirubin (Table 1). PFIC patients were 55.8% female and 44.2% male, whereas BA patients were 46.4% female and 53.6% male; the parental relationship was 81% in PFIC patients and 40% in BA patients. The majority of the pediatric patients with BA in this study (46.4%) who had liver transplants had undergone Kasei surgery prior to the procedure (almost 80%). Thirty‐six percent of the provided tissues were cadaveric, 64% were organ transplants from a living donor, and 20% were from the patients′ mothers. After a liver transplant, more than 50% of the pediatric patients who received it passed away (Table 3). Following organ transplantation, a number of causes of death were noted for the pediatric patients in our study, including multiple organ failure (21.1%), heart failure (15.8%), infection (15.8%), liver transplant rejection (10.5%), massive gastrointestinal hemorrhage (5.3%), and hepatic encephalopathy (5.3%). This study found that about 61% of the participants passed away in this manner. The average death date for these individuals was 23 months following the onset of the first symptoms. They had an average age of 4.44 ± 2.07 years and 54.9% were female. These pediatric patients reported causes of death included hepatic encephalopathy (21.6%), massive GI bleeding (11.8%), heart failure (7.8%), and infection (7.8%). In the cohort, a sample of 43 pediatric patients with PFIC was gathered. PFIC patients differed significantly from the healthy group in every liver function test. In total, 32.6% of these patients died. There were 85.7% hospital deaths and the remaining ones occurred at home. They had an average age of 4.54 ± 1.45 years. Hepatic encephalopathy (21.4%), heart failure (21.4%), and infection (14.3%) were the three causes of death that were noted. Out of the PFIC patients examined, 20 (46.5%) had liver transplants, and one of them passed away from SBP following the procedure. A cadaver provided 60% of the tissues, whereas living people contributed 40%.

**Table 3:** Comparison of the clinical characteristics and demographics of liver transplant recipients and deceased patients.

**Liver transplant**		
	**BA**	**PFIC**
	39/84 (46.4%)	20/43 (46.5%)

Age	5.48 ± 3.07	7.4 ± 2.76
Gender		
Female	23/39 (59%)	8/20 (40%)
Male	16/39 (41%)	12/20(60%)
Weight	11.07 ± 9.12	14.19 ± 7.57
PELD	17.37 ± 8.05	16.66 ± 10.12
Education		
Illiterate	35/39 (89.7%)	18/20 (90%)
Primary school students	4/39 (10.3%)	2/20 (10%)
Relation parent		
Yes	14/39 (35.9%)	17/20 (85%)
No	25/39 (64.1%)	3/20 (15%)
Donor gender		
Female	20/39 (51.3%)	10/20 (50%)
Male	19/39 (48.7%)	10/20 (50%)
Donor Type		
Living	25/39 (64.1%)	8/20 (40%)
Cadaver	14/39 (35.9%)	12/20 (60%)
Death		
Yes	19/39 (48.7%)	3/20 (15%)
No	20/39 (51.3%)	17/20 (85%)

**Death**		
	**BA**	**PFIC**
	51/84 (60.7%)	14/43 (32.6%)

Age	4.44 ± 2.07	4.54 ± 1.45
Gender		
Female	28/51 (54.9%)	7/14 (50%)
Male	23/51 (45.1%)	7/y (50%)
Weight	8.13 ± 5.26	8.97 ± 3.32
PELD	21.80 ± 10.12	19.35 ± 11.79
Education		
Illiterate	48/51 (94.1%)	14/14 (100%)
Primary school students	3/51 (5.9%)	—
Relation parent		
Yes	19/51 (37.3%)	10/14 (71.4%)
No	32/51 (62.7%)	4/14 (28.6%)
Cause of death		
Hepatic encephalopathy	11/51 (21.6%)	3/14 (21.4%)
Heart failure	4/51 (7.8%)	3/14 (21.4%)
Infection	4/51 (7.8%)	2/14 (14.3%)
Massive GI bleeding	6/51 (11.8%)	—
Other cause	18/51 (35.3%)	5/14 (35.8%)
Unknown	8/51 (15/7%)	1/14 (7.1%)
Place of death		
Hospital	44/51 (86.3%)	12/14 (85.7%)
Home	7/51 (13.7%)	2/14 (14.3%)

*Notes:* mean values ± SD.

Abbreviations: BA, biliary atresia; PFIC; progressive familial intrahepatic cholestasis.

### 3.2. Comparison of Alterations in miRNA Expression in Patients With BA and PFIC

**Figure 2:**
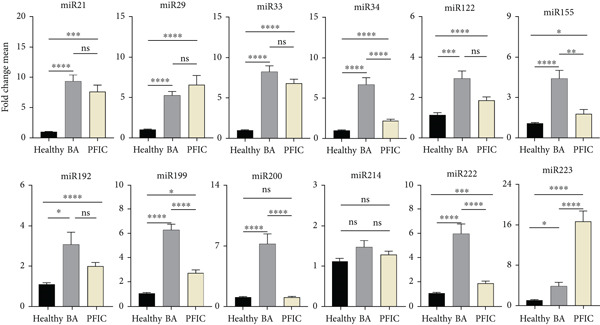
Expression variations of 12 miRNAs in BA and PFIC patients compared with healthy group. The miRNA expression changes of 50 healthy group, 43 pediatric patients with PFIC, and 84 pediatric patients with BA were compared. In comparison to the healthy group, BA and PFIC patients had increased mean expression levels of miRNAs. The findings demonstrated a significant rise in miR‐34, miR‐155, miR‐199, miR‐200, miR‐222, and miR‐223 expression in BA as compared with PFIC. miR‐223 was also significantly increased in PFIC patients compared with the BA group. The *x*‐axis shows the study groups (healthy group, BA, and PFIC); the *y*‐axis shows the fold change mean of miRNA expression; the columns indicate the bright yellow PFIC, gray BA, and black healthy group. The Livak′s method was used to calculate miRNA expression results from RT‐qPCR. The mean values ± SD are represented in the data.  ^∗^represents *p* < 0.05;  ^∗∗^represents *p* < 0.01;  ^∗∗∗^represents *p* < 0.001;  ^∗∗∗∗^represents *p* < 0.0001.

Patients with BA and PFIC had different changes in miRNA expression compared with the healthy group. Compared with PFIC patients, BA patients had higher levels of several miRNAs, including miR‐21, miR‐33, miR‐34, miR‐122, miR‐155, miR‐192, miR‐199, miR‐200b, and miR‐222. When comparing BA to the other two groups, there was a significant difference in the increase in the expression of miR‐34, miR‐155, miR‐199, miR‐200b, miR‐222, and miR‐223. Nevertheless, when PFIC pediatric patients were compared with the BA group, miR‐223 was significantly higher. Although there was an increase in miR‐29 expression in the PFIC group, it was not statistically significant (Figure 2).

#### 3.2.1. Correlation of miRNA Levels With Demographic Traits, Clinical Data, and PELD Score

The association between changes in liver indices and miRNA expression and the demographic traits of pediatric patients with BA and PFIC was assessed statistically. Although there was no correlation (*p* value> 0.05) with the other parameters assessed in this study, miRNA expression alterations were strongly correlated with the age of pediatric patients with BA and PFIC (*p* value < 0.05). The analysis of these 12 miRNAs′ expression variations using the PELD score, however, revealed a strong correlation with miR‐222 (*p* value = 0.025).

### 3.3. Levels of miRNA in PBMCs of BA Patients Compared With the Healthy Group

Pediatric patients with BA and healthy group were compared for miRNA expression levels. According to statistical analysis, of the 12 miRNAs analyzed, miR‐21, miR‐29, miR‐33, miR‐34, miR‐122, miR‐155, miR‐192, miR‐199, miR‐200b, miR‐222, and miR‐223 showed a significant difference compared with the healthy group, but no significant difference was seen for miR‐214 compared with the healthy group. The largest difference between the patient and healthy groups, as indicated by the results in Figure 2, was in miR‐21, which was eight times higher. Other miRNAs showed an increase in expression changes when compared with the healthy group, including miR‐33 [7], miR‐34 [6], miR‐199 [5], miR‐200b [6], and miR‐222 [5], miR‐29a [4], miR‐122 [2], miR‐155 [4], miR‐192 [2], and miR‐223 [3].

#### 3.3.1. Receiver Operating Characteristic (ROC) Curve and Heatmap Analysis for the Studied miRNAs in BA Patients

The ROC curves for 12 different miRNAs are displayed in Figure 3a. Compared with other miRNAs, the AUC values of miR‐21 (AUC = 0.94), miR‐29 (AUC = 0.94), miR‐33 (AUC = 0.98), miR‐199 (AUC = 0.96), and miR‐222 (AUC = 0.95) were significantly higher, indicating their increased significance as diagnostic markers in these patients. The diagnostic value of miRNA is typically assessed using ROC and AUC, with an AUC near 1 indicating strong diagnostic power [15]. The R software generated a heatmap graphic that clearly distinguished the groups under study, using data from variations in miRNA expression between the healthy group and BA groups (Figure 3b).

Figure 3The ROC curves and heatmap related to the analysis of the expression changes of 12 miRNAs in healthy group pediatric patients with BA. (a) The ROC curves are used to evaluate the diagnostic ability of studied miRNAs in distinguishing BA patients from the control group. The ROC curve is shown in black. The 95% ROC confidence interval is represented by the red curve. Significant diagnostic potential is indicated by the AUC values of miR‐21, miR‐29, miR‐33, miR‐199, and miR‐222. (b) Heatmap—the heatmap displays the relative expression patterns and divides samples from the healthy group and patients with PFIC into groups of 12 miRNAs. The relative expression of miRNAs is represented by color according to the intensity of expression (increase or decrease).

**Figure:**
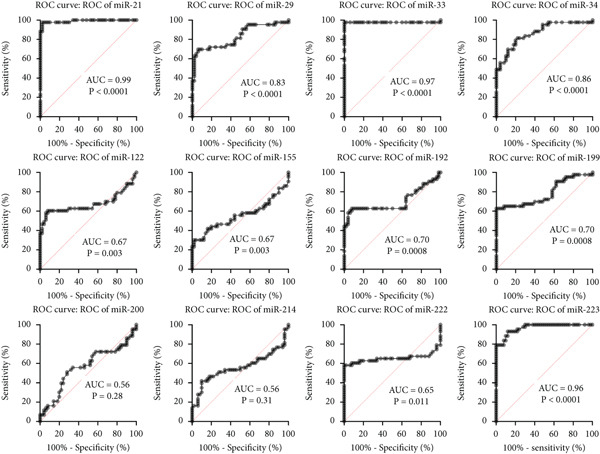
(a)

**Figure:**
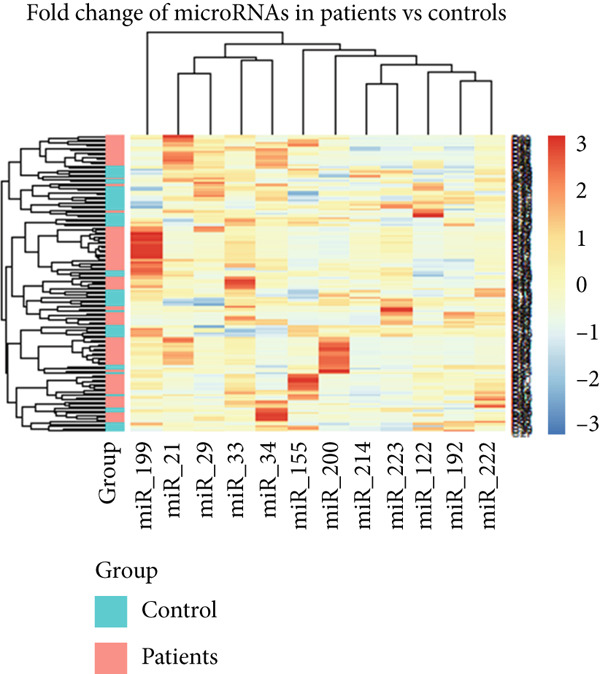
(b)

#### 3.3.2. Association Between miRNA Expression Levels and Liver Transplant in BA Patients

**Figure 4:**
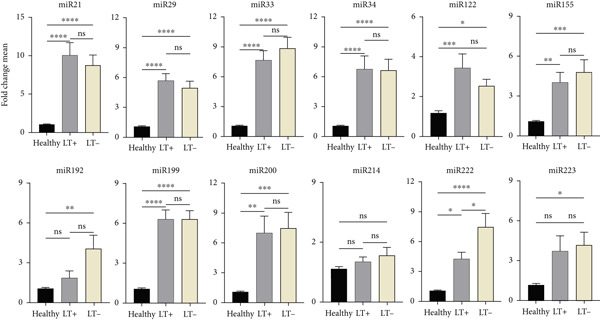
Comparison of serum miR profiling changes in transplanted (LT+) and nontransplanted (LT−) BA patients with the healthy group. Changes in the expression of 12 microRNAs were examined between the control group and the two groups of the 84 BA patients, 39 of whom received liver transplantation and 45 of whom did not. There was only a significant change in miR‐222 when comparing the LT− and LT+ groups. *y*‐axis: fold change means in miRNA expression; *x*‐axis: study groups (healthy group, LT−, and LT+); colors: healthy group (black), LT+ (gray), and LT− (light yellow). ns: nonsignificant;  ^∗^represents *p* < 0.05;  ^∗∗^represents *p* < 0.01;  ^∗∗∗^represents *p* < 0.001;  ^∗∗∗∗^represents *p* < 0.0001.

Statistical analysis revealed variations in the levels of the examined miRNAs among 39 pediatric patients with BA who had liver transplantation. Some of these miRNAs (miR‐33, miR‐155, miR‐192, miR‐200, and miR‐222) had decreased expression, whereas others (miR‐21, miR‐29, miR‐34, and miR‐122) had increased expression. However, miR‐222 expression was significantly lower compared with the healthy group. This miRNA may be introduced as a marker indicating the need for liver transplantation (Figure 4). It seems that there is a need for a larger number of patients to accurately determine this relationship.

#### 3.3.3. Association Between miRNA Expression Levels and Death of BA Patients

**Figure 5:**
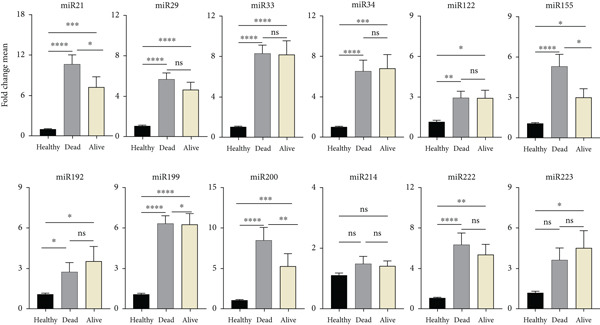
Comparison of serum miR profiling changes in the patient groups: survivors (alive) and deceased (dead) with the healthy group. When compared with the healthy group and patients who survived, the levels of miR‐21, miR‐155, miR‐199, and miR‐200 in the group of deceased patients were significantly higher. *y*‐axis: average fold change in miRNA expression; *x*‐axis: research groups (healthy group, alive, and dead); colors: blue for healthy group, light yellow for alive, and gray for death. ns: nonsignificant;  ^∗^represents *p* < 0.05;  ^∗∗^represents *p* < 0.01;  ^∗∗∗^represents *p* < 0.001,  ^∗∗∗∗^represents *p* < 0.0001.

The relationship between miRNA changes in BA patients and their death was evaluated statistically. In the statistical analysis, it was found that some miRNAs in patients who died had increased expression compared with living patients (Figure 5). miR‐21, miR‐155, miR‐199, and miR‐200 in BA patients showed a significant increase compared with the healthy group and patients who survived (*p* value < 0.05). This increased expression can probably be considered a biological marker in these patients.

### 3.4. Levels of miRNA in PBMCs of PFIC Patients Compared With the Healthy Group

Pediatric patients with PFIC and healthy group were compared for miRNA expression levels. According to statistical analysis, of the 12 miRNAs analyzed, miR‐21, miR‐29, miR‐33, miR‐34, miR‐122, miR‐155, miR‐192, miR‐199, miR‐222, and miR‐223 showed a significant difference, whereas two (miR‐200b and miR‐214) showed an increase when compared with the healthy group, but no significant difference was observed. The largest difference between the patient and healthy groups, as indicated by the results in Figure 2, was in miR‐223, which was 14 times higher. Moreover, there was an almost six‐fold increase in the miRNAs miR‐21, miR‐29, and miR‐33.

#### 3.4.1. ROC Curves and Heatmap Analysis for the Studied miRNAs in PFIC Patients

ROC curves are shown for twelve distinct miRNAs in Figure 6a. In these individuals, miR‐21 (AUC = 0.97), miR‐33 (AUC = 0.97), miR‐223 (AUC = 0.94), miR‐34 (AUC = 0.81), and miR‐29 (AUC = 0.80) had significantly higher AUC values than other miRNAs, suggesting their greater relevance as diagnostic indicators. Utilizing information from differences in miRNA expression between the healthy group and PFIC groups, the heatmap figure was produced. The heatmap graph showed significant differences between the groups being studied (Figures 6b).

Figure 6The ROC curves and heatmap related to the analysis of the expression changes of 12 miRNAs in pediatric patients with PFIC. (a) ROC curves are used to evaluate the diagnostic ability of studied miRNAs in distinguishing PFIC patients from the controls. Significant diagnostic potential is indicated by the AUC values of miR‐21, miR‐223, miR‐33, miR‐34, and miR‐29. The ROC curve is shown in black. The 95% ROC confidence interval is represented by the red curve. The diagonal is shown by the blue line. (b) Heatmap—the heatmap displays the relative expression patterns and divides samples from the healthy group and patients with PFIC into groups of 12 miRNAs. The relative expression of miRNAs is represented by color according to the intensity of expression (increase or decrease).

**Figure:**
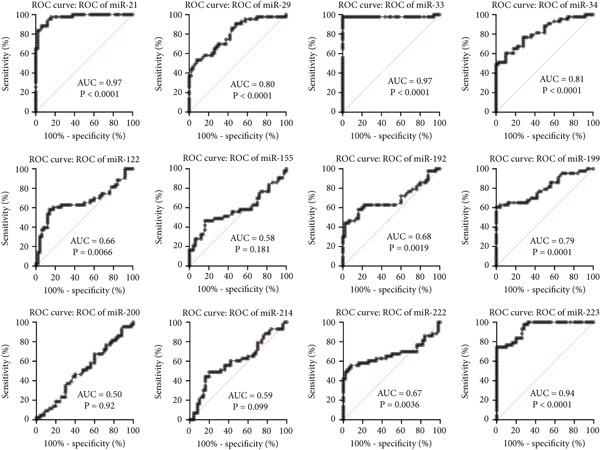
(a)

**Figure:**
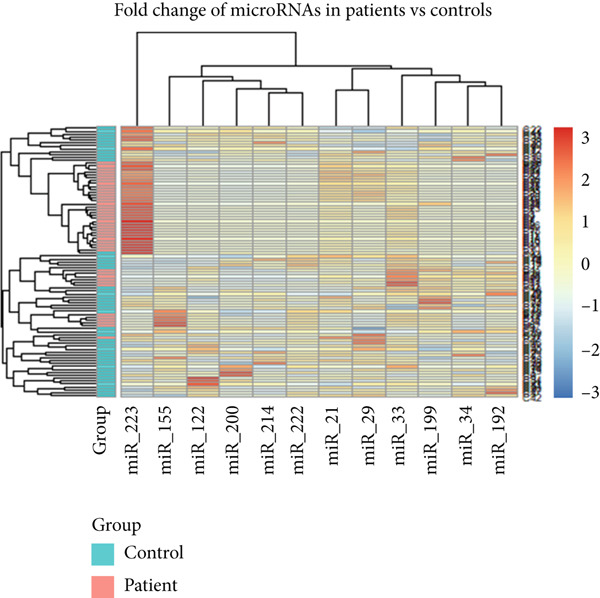
(b)

#### 3.4.2. Relationship Between PFIC Patients′ Liver Transplants and miRNA Expression Levels

**Figure 7:**
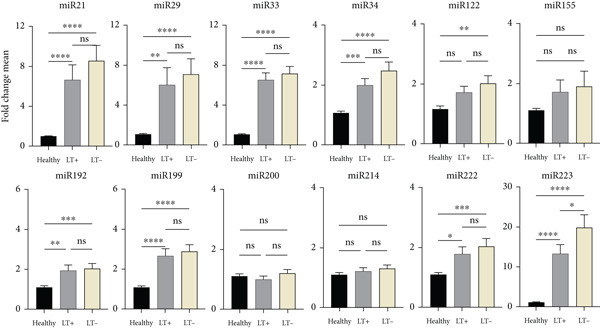
Comparison of serum miR profiling changes in transplanted (LT+) and nontransplanted (LT−) PFIC patients with the healthy group. When comparing changes in miRNA expression between the LT+ and LT− groups, no significant distinction was observed. *y*‐axis: fold change means in miRNA expression; *x*‐axis: study groups (healthy group, LT−, and LT+); colors: healthy group (black), LT+ (gray), and LT− (light yellow). ns: nonsignificant;  ^∗^represents *p* < 0.05;  ^∗∗^represents *p* < 0.01;  ^∗∗∗^represents *p* < 0.001;  ^∗∗∗∗^ represents *p* < 0.0001.

The majority of the miRNAs that were examined showed statistically significant differences in expression between the liver‐transplanted PFIC patients and the nontransplant group, with the exception of miR‐223 (Figure 7).

#### 3.4.3. Relationship Between PFIC Patients′ Deaths and miRNA Expression Levels

**Figure 8:**
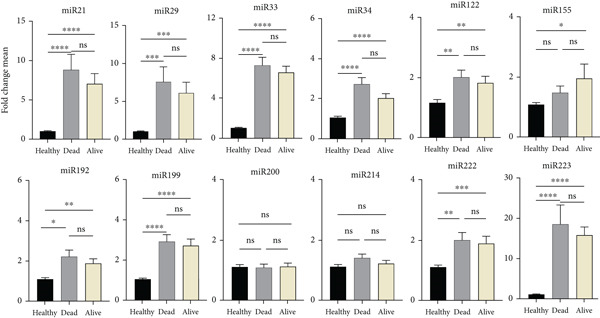
Comparison of serum miR profiling changes in the PFIC patient groups: survivors (alive) and deceased (dead) with the healthy group. When compared with the healthy group and patients who survived the level of miR‐34 in the deceased patient group was significantly higher. *y*‐axis: average fold change in miRNA expression; *x*‐axis: research groups (healthy group, alive, and dead); colors: blue for healthy group, light yellow for alive, and gray for death. ns: nonsignificant,  ^∗^represents *p* < 0.05;  ^∗∗^represents *p* < 0.01,  ^∗∗∗^represents *p* < 0.001,  ^∗∗∗∗^represents *p* < 0.0001.

PFIC patients who died had higher expression of most of the miRNAs that were analyzed, including miR‐21, miR‐223, miR‐33, miR‐34, and miR‐29, than those who survived. A decrease was also observed in certain miRNAs, such as miR‐155. Between the groups of deceased and living patients and the healthy group, miR‐200 and miR‐214 did not significantly differ. miR‐34 was the only one that significantly increased (Figure 8).

### 3.5. Evaluating the Association Between the Studied miRNAs and Liver Cirrhosis Using Bioinformatics Tools

In order to understand the pathophysiology of fibrosis and cirrhosis, it is crucial to identify the signaling pathways and significant miRNAs in gene expression. Determining the target genes of miRNAs is essential for this reason, in addition to analyzing the alterations in their expression as crucial molecules in controlling gene expression. Since there are not many studies on the involvement of miRNAs in PFIC, we focused on how important the miRNAs under study are in controlling gene expression in signaling pathways that cause cirrhosis in BA patients. Finding the significance of these miRNAs in cholestatic individuals′ liver cirrhosis was the goal (Table 4).

**Table 4:** The target genes and downstream signaling pathways of the studied miRNAs.

**miRNA**	**Etiology**	**Gene**	**Signaling pathway**	**Outcome activation HSCs**	**Ref.**
miR‐21‐5p	BA	PTEN	PI3K/AKT	*α-SMA*	[16, 17]
	Liver fibrosis	PTEN	PI3K/AKT	*α-SMA, Col1A1*	[18]
miR‐29a‐3p	BA	IGF‐1, Il1RAP	PI3K/AKT	*Col1A1*	[19]
	Liver fibrosis	IGF‐1, PI3KR1, LOX, PDGF‐C	PI3K/AKT		[20]
miR‐33a‐3p	BA	Not reported			
	Liver fibrosis	TGF‐*β*1	PI3K/AKT	*Col1A1, α-SMA*	[21]
miR‐222‐3p	BA	PPP2R2A, IGF‐1	PI3K/AKT	*Col1A1, α-SMA*	[22–24]
	Liver fibrosis	CDKN1B	PI3K/AKT	*Col1A1, α-SMA*	[25]
miR‐200b‐3p	BA	PTEN	PI3K/AKT	*MMP-2*	[26]
	Liver fibrosis	TGF‐*β*1	TGF‐*β* signaling	*α-SMA, Col1A1*	[27]
miR‐122‐5p	BA	Not reported			
	Liver fibrosis	TGF‐*β*1	TGF‐*β* signaling	*α-SMA, E-cadherin,*	[28]
miR‐155‐5p	BA	Not reported			
	Liver fibrosis	TGF‐*β*1, Smad2/3	TGF‐*β* signaling	*α-SMA, MMP2, MMP9, MMP12, TIMP1*	[29]
miR‐223‐3p	BA	Not reported			
	Liver fibrosis	Smad2/3	TGF‐*β* signaling	*α-SMA*	[30]
miR‐199a‐5p	BA	Not reported			
	Liver fibrosis	SIRT1	TGF‐*β* signaling	*α-SMA, Col1A1*	[31]
miR‐34a‐5p	BA	Not reported			
	Liver fibrosis	PPAR*γ*, TGF‐*β*1, Smad2/3, SIRT1	TGF‐*β* signaling PPAR‐*γ* signaling SIRT1/p53	*α-SMA*	[32–35]
miR‐192‐5p	BA	Not reported			
	Liver fibrosis	Smad4, Smad2/3, Akt, Rictor	TGF‐*β* signaling AKT/mTORC		[36]
miR‐214‐5p	BA	SUFU	Hedgehog signaling pathway		[37]
	Liver fibrosis	SUFU	Hedgehog signaling pathway	*α-SMA, Col1A1*	[38]

Abbreviations: *α-SMA*, alpha‐smooth muscle actin; *CDKN1B*, cyclin‐dependent kinase inhibitor 1B; *PPP2R2A*, serine/threonine‐protein phosphatase 2A; *PTEN*, phosphatase and tensin homolog; *Smad*, suppressor of mothers against decapentaplegic; *TGF-β*, transforming growth factor‐*β*.

To determine the involvement of miRNAs and genes in fibrosis and cirrhosis, a gene network was built using previous research and the current investigation′s results. Gene network results suggest that some genes, including *Smad2/3, SIRT1, PTEN, TGF-β1,* and *IGF-1*, may play a more significant role in the fibrosis process in BA patients. The pathophysiology of fibrosis in these patients may be more dependent on the activation of these genes, which appear to be controlled by a number of significant miRNAs in liver fibrosis (Figure 9). The examined miRNAs were shown to be crucial in controlling liver fibrosis in BA patients based on the gene network created using enrichment analysis in this paper and a review of the references (Table 4). The two signaling pathways that the miRNAs under investigation seem to be activating to promote liver fibrosis and eventually cirrhosis are the PI3K/AKT and TGF‐*β* signaling pathways.

Figure 9A significant regulatory network of genes and miRNAs in patients. (a) The gene network designs demonstrate the interactions between the miRNAs under study and the genes that are predicted to be their targets. Green nodes (squares) represent miRNAs, blue nodes (circles) represent target genes, and the connecting lines represent regulatory interactions between them. (b) Using the KEGG database, the signaling pathways associated with the target genes were identified. Fibrosis and cellular metabolic processes were within the key pathways, which also had an impact on the TGF‐*β* and PI3K/AKT signaling pathways. According to this analysis, the miRNAs under investigation may be crucial in controlling molecular pathways associated with liver damage and recovery. *CDKN1B*, cyclin‐dependent kinase inhibitor 1B; *TGF-β*, transforming growth factor‐*β*; *Smad*, suppressor of mothers against decapentaplegic; *PPP2R2A*, serine/threonine‐protein phosphatase 2A; *α-SMA*, alpha‐smooth muscle actin; *PTEN*, phosphatase and tensin homolog.

**Figure:**
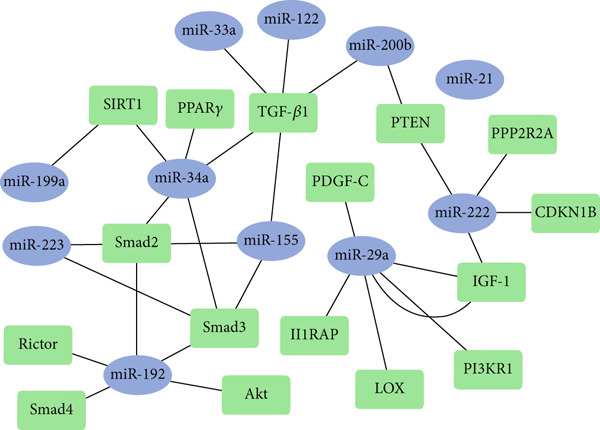
(a)

**Figure:**
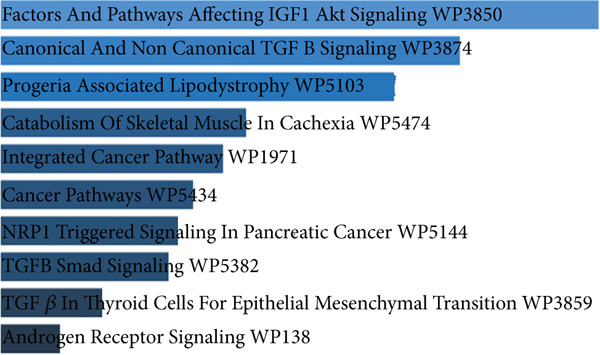
(b)

## 4. Discussion

**Figure 10:**
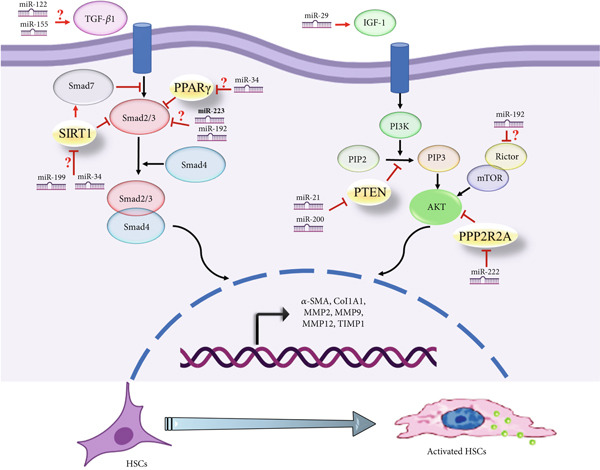
This figure shows the investigated miRNAs′ functional role in the progression of liver fibrosis in cirrhotic patients. Changes in miRNA expression in patients activate the main molecular pathways indicated in this graphic. Two key signaling pathways have been demonstrated to be crucial interactions in the progression of fibrosis: PI3K/AKT and TGF‐*β*. The investigated miRNAs regulate important elements of these pathways, such as genes involved in cell proliferation, cell differentiation, and extracellular matrix synthesis, that regulate the development and progression of liver fibrosis. *TIMPs*, tissue inhibitors of metalloproteinases; *CDKN1B*, cyclin‐dependent kinase inhibitor 1B; *TGF-β*, transforming growth factor‐*β*; *Smad*, suppressor of mothers against decapentaplegic; *PPP2R2A*, serine/threonine‐protein phosphatase 2A; *α-SMA*, alpha‐smooth muscle actin; *PTEN*, phosphatase and tensin homolog; *MMP*, matrix metalloproteinase.

Pediatric liver cirrhosis and fibrosis are serious clinical conditions that can result in liver failure, leading to a transplant. Recent data indicates that miRNAs are essential for the regulation of molecular processes associated with fibrosis, apoptosis, and inflammation. These findings imply that the identification of potential biomarkers and the further clarification of pathological mechanisms could be achieved by investigating miRNA expression patterns in relatives with liver diseases. The expression changes of a collection of miRNAs have been assessed in patients with BA and PFIC to assess their correlation with the severity of the disease and the fibrosis process. The results indicated that pediatric patients with cirrhosis caused by BA and PFIC etiologies have different patterns of miRNA expression throughout their bodies. Significantly higher amounts of miR‐34, miR‐155, miR‐199, miR‐200b, and miR‐222 were found in BA patients than in other individuals. The expression level of miR‐223 was significantly different in patients with PFIC etiology. Although cirrhosis is the final stage of liver disorders, variations in the healthy group of miRNA expression suggest different pathways that could contribute to the evolution of the fibrosis process and cirrhosis in liver diseases. It can be investigated as a consideration in miRNA therapy and is significant for both diagnosis and prognosis. According to the bioinformatics analysis conducted for the present study, every studied miRNA has a significant impact on the fibrosis process. The involvement of these miRNAs in liver fibrosis and cirrhosis is shown in Figure 10. The expression changes of these miRNAs cause fibrosis by activating HSCs. The importance of these miRNAs in BA and PFIC cirrhosis is the main focus of this study, even though the data indicate that all of the miRNAs examined have a significant function in pediatric liver cirrhosis and have high study value. Liver fibrosis is generally caused by the activation of HSC cells via a variety of signaling pathways, including WNT/*β*‐catenin, TGF‐*β*, PI3K/AKT, Hedgehog, and PPAR‐*γ* [39, 40].

According to earlier reports, BA patients have elevated levels of miR‐200b‐3p and miR‐21, which are consistent with the results of this study. *PTEN* is the target gene of these two miRNAs. The suppression of the PI3K/AKT signaling pathway is caused by *PTEN*. Thus, the advancement of liver fibrosis is facilitated by the elevated expression of these two miRNAs. These two miRNAs are being considered as potential therapeutics due to their significance. In BA patients, miR‐200b was reported to upregulate matrix metalloproteinase‐2 (*MMP-2*) expression [41]. CircCHD2 was identified as a miR‐200b‐3p sponge that suppressed *Col1A1* and reduced *α-SMA* expression, demonstrating the significance of this miRNA in liver fibrosis [27]. Interestingly, miR‐200 was one of the miRNAs that varied between BA and PFIC cholestatic in this study; however, it did not significantly differ in PFIC patients when compared with the healthy group. It would be a good opportunity for additional research because these results should be examined in a larger patient population. Additionally, pediatric patients with BA exhibited a substantial upregulation of miR‐222‐3p. The results of other researchers were in agreement with these findings. In BA patients, miR‐222‐3p was found to reduce the *PPP2R2A* activity, which in turn suppresses Akt, causing the PI3K/Akt fibrosis pathway to become activated [22]. Using long noncoding RNA growth arrest‐specific 5 (lncRNA GAS5), an inhibitor of this miR, researchers observed that fibrosis was decreased in BA, highlighting the significance of miR‐222‐3p in the treatment of fibrosis in BA [24].

This study indicated that miR‐155‐5p had elevated expression. By activating the TGF‐*β*1 and TGF‐*β* signaling pathways, miR‐155‐5p promotes hepatic fibrosis [28, 29]. There have been no reports of this mechanism in BA patients to date. The TGF‐*β* signaling pathway may also be triggered in these patients by raising the expression of miR‐155‐5p, which ultimately leads to cirrhosis in these pediatric patients, as our results showed that miR‐155‐5p was significantly elevated in pediatric patients with BA. However, future research requires evaluating the active proteins using alternative techniques. BA patients in this study had elevated levels of two miRNAs: miR‐34a‐5p and miR‐199a‐5p. It is unclear how these two miRNAs relate to BA pediatric patients′ fibrosis. However, according to reports, *SIRT1* is their target gene during the fibrosis process. Through acetylation, SIRT1 has been demonstrated to decrease *p53* function in the context of miR‐34a‐5p. When *SIRT1* is inhibited by miR‐34a‐5p, p53 is activated, which leads to apoptosis [33, 34]. Because cholestasis from common bile duct blockage occurred in hepatic fibrosis mice, *SIRT1* was downregulated, and miR‐199a‐5p was upregulated. Although *SIRT1* suppressed the expressions of *TGF-β1, p-Smad3,* and *α-SMA*, it was found to boost the expression of Smad7 [31]. These results have also been documented in hepatic fibrosis in rhesus monkeys [42]. miR‐34a‐5p has been demonstrated to induce fibrosis by inhibiting *PPARγ*. In the TGF‐*β* signaling pathway, *PPARγ* suppresses the transcription factors Smad2/3 [32]. Further investigation is required to determine which of these signaling pathways is triggered in BA pediatric patients by the higher expression of miR‐34a‐5p and miR‐199a‐5p. Our results demonstrated that PFIC has substantially greater alterations in miR‐223 expression than BA, and that these alterations appear to be crucial in the development of liver cirrhosis in pediatric patients with PFIC etiology. It may, however, serve as a diagnostic tool for PFIC and BA if additional research shows comparable results. The function of miR‐223 in PFIC is not described. The crucial role that miR‐223 plays in liver inflammation and fibrosis, however, makes the significance of this miRNA in PFIC illness intriguing, contentious, and possibly difficult to understand. According to some research, miR‐223 may regulate inflammatory responses and has a regulatory function in immune cells, despite indications of a protective role in liver disorders [43, 44]. Therefore, PFIC and BA patients′ elevated blood levels of miR‐223 may be a compensatory mechanism for regulating inflammation. However, the results demonstrated that the expression of this miRNA varied with the phases of fibrosis, with the expression of miR‐223‐3p increasing in the latter stages of liver fibrosis. According to their results, cirrhosis (F4) and severe fibrosis (≥ F3) had significantly higher serum levels of miR‐223 than F0‐F2 and F0‐F3, respectively [45]. Since it appears that the miRNA level rises as fibrosis and cirrhosis progress, these researchers thought that variations in this miRNA expression were crucial for identifying various stages of fibrosis and proposed that these miRNAs might be used as biomarkers of the course of the disease. miR‐223‐3p expression also significantly increased in the current study, suggesting an important role for this miRNA in pediatric cirrhosis.

The most critical therapy option for pediatric patients with cholestasis, particularly BA and PFIC, is liver transplantation [46]. Similar results from previous researchers in this field and our study′s high liver transplantation rate (about 50% for BA and PFIC) underscore the continued difficulties in treating pediatric patients with cholestasis. Even with improvements in liver transplantation strategies, such as primary liver transplantation prior to the Kasai procedure, and new methods and therapies to improve survival in these pediatric patients, post‐transplant medication changes, donor type ratios, and postliver transplant mortality rates are still high [47]. Our study participants′ mortality statistics revealed that, whereas just 33% of PFIC pediatric patients died, over 61% of BA pediatric patients did. Lemoine et al.′s study found that almost half of BA infants who receive liver transplants pass away within 2 years of the procedure [48]. Heart failure and hepatic encephalopathy were the leading causes of death for PFIC patients and hepatic encephalopathy for BA patients in our study. The prognosis of cholestasis patients may be affected by the 12 miRNAs we examined, some of which were substantially linked to liver transplantation and pediatric mortality. For BA patients, miR‐222 had a significant relationship with both liver transplantation and the PELD score; however, for PFIC pediatric patients, no significant correlation was found between the miRNAs under investigation, liver transplantation, and the PELD score. When it came to the relationship between mortality in pediatric patients with cholestasis and variations in miRNA expression, miR‐21, miR‐155, miR‐199, and miR‐200 were shown to be strongly correlated with death in BA patients, whereas miR‐34 was significantly correlated with death in PFIC patients. Although there are not many studies that analyze the variations in miRNA expression among the causes of cholestasis, one study found that miR‐21 in mice was a unique indicator of BA in contrast to other cholestatic illnesses [49]. Based on the results of this study, it may be feasible to various between different forms of cholestasis by examining miRNAs. Compared with other disorders, these results are promising since they increase the likelihood of finding miRNAs unique to a given disease. Nonetheless, it is evident that more investigation is required. The results of comparing changes in miRNA levels in patients with the healthy group and AUC values suggest that some of the miRNAs that have been studied, such as miR‐21, miR‐29, miR‐33, miR‐199, and miR‐222, in BA patients and miR‐21, miR‐223, miR‐33, miR‐34, and miR‐29, in PFIC patients, may be helpful for prognosis.

The significance of these miRNAs in BA patients is demonstrated by these studies. Patients benefit greatly from early diagnosis since pediatric patients who undergo treatment have a higher probability of recovering, possibly as a result of reduced fibrosis or inflammation. In these conditions, it is difficult to make a clinical diagnosis using techniques like endoscopic retrograde cholangiopancreatography or destructive liver biopsy. Currently, scientists have emphasized the use of miRNAs as noninvasive indicators for the early detection of this disease. Therefore, comparing additional miRNAs at different phases of these diseases could be a very useful recommended study to help guide the prognosis of cholestatic patients, even if it could help identify the cause. Alternatively, a comprehensive evaluation of these miRNAs′ gene expression alterations may yield detailed insights into the disease′s pathophysiology. A limitation of this research was the shortage of patient follow‐up samples. Although the analysis of miRNAs, particularly unreported miRNAs, produced useful data, more thorough information on the function and significance of these miRNAs in patients may be published if the expression changes of these miRNAs are compared in follow‐up samples.

## 5. Conclusion

Based on the comparison of the two samples, it was determined that there are significant variations in the expression of miRNAs in the BA and PFIC samples. Additionally, the ROC curve′s AUC values demonstrated the diagnostic role of different miRNAs in BA and PFIC. Significant correlations were identified between changes in miRNA expression and liver transplantation and pediatric mortality in BA and PFIC. These miRNAs may play a fibrotic role in liver cirrhosis, particularly in the TGF‐*β* and PTEN/PI3K/Akt signaling pathways. Thus, the results highlight the significance of these miRNAs in liver cirrhosis in pediatric patients with cholestasis symptoms. On the other hand, their differential expression in BA and PFIC may have diagnostic and therapeutic significance and suggests that distinct molecular pathways are activated in the pathophysiology of these individuals. Determining the significance of these miRNAs in the disease and identifying viable treatment options for liver cirrhosis seem to depend on more research in cholestatic patients.

**Nomenclature**
*α*‐SMA: alpha‐smooth muscle actin
BA: biliary atresia
CDKN1B: cyclin‐dependent kinase inhibitor 1B
ECM: extracellular matrix
IGF‐1: insulin‐like growth factor 1
KPE: Kasai portoenterostomy
lncRNA: long noncoding RNA
MMPs: matrix metalloproteinases
miRNA: microRNA
PPP2R2A: Serine/threonine‐protein phosphatase 2A
PFIC: progressive familial intrahepatic cholestasis
PI3K/AKT: phosphoinositide 3‐kinase/protein kinase B
PTEN: Phosphatase and tensin homolog
PPAR‐*γ*
: peroxisome proliferator‐activated receptor *γ*
ROC: receiver operating characteristic
SIRT1: sirtuin 1
Smad: suppressor of mothers against decapentaplegic
TIMPs: tissue inhibitors of metalloproteinases
TGF‐*β*
: transforming growth factor‐*β*

## Disclosure

All authors have read and agreed to the published version of the manuscript.

## Conflicts of Interest

The authors declare no conflicts of interest.

## Author Contributions

Conceptualization: F.K. and R.Y.; methodology: F.K., R.Y., and A.A.; software: F.K.; validation: F.K., R.Y., A.A., N.M., B.G., T.K., and S.M.D.; formal analysis: F.K., A.A., and R.Y.; investigation: F.K. and R.Y.; resources: F.K., R.Y., N.M., B.G., T.K., and S.M.D.; data curation: F.K., R.Y., N.M, B.G, T.K., and S.M.D.; writing—original draft preparation: F.K. and R.Y.; writing—review and editing: F.K. and R.Y.; visualization: F.K. and R.Y.; supervision: R.Y.; project administration: R.Y.; funding acquisition: R.Y.

## Funding

This study was supported by the Shiraz University of Medical Sciences, 10.13039/501100004320, 27218.

## Data Availability

The data are available from the corresponding author upon request.

## References

[1] Mawardi M. , Alalwan A. , Fallatah H. , Abaalkhail F. , Hasosah M. , Shagrani M. , Alghamdi M. Y. , and Alghamdi A. S. , Cholestatic Liver Disease, Journal of Gastroenterology. (2021) 27, no. Supplement 1, S1–S26, 10.4103/sjg.sjg_112_21.

[2] Zhang B. P. , Huang Z. H. , and Dong C. , Biliary Atresia Combined With Progressive Familial Intrahepatic Cholestasis Type 3: A Case Report and Review of the Literature, Medicine (Baltimore). (2019) 98, no. 19, e15593, 10.1097/md.0000000000015593, 2-s2.0-85066060599, 31083246. 31083246 PMC6531222

[3] Tam P. K. H. , Wells R. G. , Tang C. S. M. , Lui V. C. H. , Hukkinen M. , Luque C. D. , De Coppi P. , Mack C. L. , Pakarinen M. , and Davenport M. , Biliary Atresia, Nature Reviews Disease Primers. (2024) 10, no. 1, 10.1038/s41572-024-00533-x. PMC11956545 38992031

[4] Nomden M. , Beljaars L. , Verkade H. J. , Hulscher J. B. F. , and Olinga P. , Current Concepts of Biliary Atresia and Matrix Metalloproteinase-7: A Review of Literature, Frontiers in Medicine (Lausanne). (2020) 7, 617261, 10.3389/fmed.2020.617261, 33409288. PMC7779410 33409288

[5] Amirneni S. , Haep N. , Gad M. A. , Soto-Gutierrez A. , Squires J. E. , and Florentino R. M. , Molecular Overview of Progressive Familial Intrahepatic Cholestasis, World Journal of Gastroenterology. (2020) 26, no. 47, 7470–7484, 10.3748/wjg.v26.i47.7470, 33384548. 33384548 PMC7754551

[6] Pinto R. B. , Schneider A. C. , and da Silveira T. R. , Cirrhosis in Children and Adolescents: An Overview, Journal of Hepatology. (2015) 7, no. 3, 392–405, 10.4254/wjh.v7.i3.392, 2-s2.0-84928949937, 25848466. PMC4381165 25848466

[7] Sun G. R. and Burns M. , Progressive Familial Intrahepatic Cholestasis: A Rare Cause of Cirrhosis in Young Adult Patients, Case Reports in Medicine. (2015) 2015, 428638, 10.1155/2015/428638, 2-s2.0-85032643595. 26136783 PMC4468343

[8] Zhou W. C. , Zhang Q. B. , and Qiao L. , Pathogenesis of Liver Cirrhosis, World Journal of Gastroenterology. (2014) 20, no. 23, 7312–7324, 10.3748/wjg.v20.i23.7312, 2-s2.0-84902668983. 24966602 PMC4064077

[9] Smith M. , Zuckerman M. , Kandanearatchi A. , Thompson R. , and Davenport M. , Using Next-Generation Sequencing of MicroRNAs to Identify Host and/or Pathogen Nucleic Acid Signatures in Samples From Children With Biliary Atresia—A Pilot Study, Access Microbiology. (2020) 2, no. 7, acmi000127, 10.1099/acmi.0.000127, 32974591. 32974591 PMC7497833

[10] Ho P. T. B. , Clark I. M. , and Le L. T. T. , MicroRNA-Based Diagnosis and Therapy, International Journal of Molecular Sciences. (2022) 23, no. 13, 10.3390/ijms23137167. PMC9266664 35806173

[11] Motazedian N. , Geramizadeh B. , Dehghani S. M. , Azarpira N. , Hossein Aghdaei M. , Yaghobi R. , Shamsaeefar A. , Kazemi K. , Karimi M. H. , Mirahmadizadeh A. , Mashhadiagha A. , Ataollahi M. , Ilkhanipoor H. , Basiratnia M. , Nemati H. , Ekramzadeh M. , Sanaei Dashti A. , Nikeghbalian S. , and Malekhosseini S. A. , Cohort Profile: Shiraz Pediatric Liver Cirrhosis Cohort (SPLCCS), Archives of Iranian Medicine. (2023) 26, no. 4, 229–233, 10.34172/aim.2023.35, 38301084. 38301084 PMC10685747

[12] Lak R. , Yaghobi R. , and Garshasbi M. , Importance of miR-125a-5p and miR-122-5p Expression in Patients With HBV Infection, Cellular and Molecular Biology (Noisy-le-Grand). (2020) 66, no. 5, 1–8, 10.14715/cmb/2020.66.5.1. 33040804

[13] Abdolyousefi E. N. , Motalleb G. , and Yaghobi R. , Association Between ACOX1 and NRF1 Gene Expression and Hepatitis B and C Virus Infections and Hepatocellular Carcinoma in Liver Transplant Patients (Shiraz, Iran), Transplantation. (2022) 20, no. 1, 52–58, 10.6002/ect.2021.0175. 34763625

[14] Afshari A. , Yaghobi R. , Karimi M. H. , and Mowla J. , Alterations in MicroRNA Gene Expression Profile in Liver Transplant Patients With Hepatocellular Carcinoma, BMC Gastroenterology. (2021) 21, no. 1, 10.1186/s12876-020-01596-2, 34118888. PMC8199419 34118888

[15] Li R. , Lu C. , Yang W. , Zhou Y. , Zhong J. , Chen X. , Li X. , Huang G. , Peng X. , Liu K. , Zhang C. , Hu H. , and Lai Y. , A Panel of Three Serum MicroRNA Can Be Used as Potential Diagnostic Biomarkers for Nasopharyngeal Carcinoma, Journal of Clinical Laboratory Analysis. (2022) 36, no. 2, e24194, 10.1002/jcla.24194. 35028969 PMC8842135

[16] Makhmudi A. , Kalim A. S. , and Gunadi , MicroRNA-21 Expressions Impact on Liver Fibrosis in Biliary Atresia Patients, Notes. (2019) 12, no. 1, 10.1186/s13104-019-4227-y, 2-s2.0-85063744643. PMC6441216 30925941

[17] Shen W. , Chen G. , Dong R. , Zhao R. , and Zheng S. , MicroRNA-21/PTEN/Akt Axis in the Fibrogenesis of Biliary Atresia, Journal of Pediatric Surgery. (2014) 49, no. 12, 1738–1741, 10.1016/j.jpedsurg.2014.09.009, 2-s2.0-84915747895, 25487473. 25487473

[18] Xue J. , Xiao T. , Wei S. , Sun J. , Zou Z. , Shi M. , Sun Q. , Dai X. , Wu L. , Li J. , Xia H. , Tang H. , Zhang A. , and Liu Q. , miR‐21‐Regulated M2 Polarization of Macrophage Is Involved in Arsenicosis-Induced Hepatic Fibrosis Through the Activation of Hepatic Stellate Cells, Journal of Cellular Physiology. (2021) 236, no. 8, 6025–6041, 10.1002/jcp.30288, 33481270. 33481270

[19] Hand N. J. , Horner A. M. , Master Z. R. , Boateng L. A. , LeGuen C. , Uvaydova M. , and Friedman J. R. , MicroRNA Profiling Identifies miR‐29 as a Regulator of Disease-Associated Pathways in Experimental Biliary Atresia, Journal of Pediatric Gastroenterology and Nutrition. (2012) 54, no. 2, 186–192, 10.1097/MPG.0b013e318244148b, 2-s2.0-84856258714, 22167021. 22167021 PMC3264748

[20] Jiang X. P. , Ai W. B. , Wan L. Y. , Zhang Y. Q. , and Wu J. F. , The Roles of MicroRNA Families in Hepatic Fibrosis, Cell & Bioscience. (2017) 7, no. 1, 10.1186/s13578-017-0161-7, 2-s2.0-85021686741. PMC5496266 28680559

[21] Li Z.-J. , Ou-Yang P.-H. , and Han X.-P. , Profibrotic Effect of miR-33a With Akt Activation in Hepatic Stellate Cells, Cellular Signalling. (2014) 26, no. 1, 141–148, 10.1016/j.cellsig.2013.09.018, 2-s2.0-84887187262. 24100264

[22] Shen W. J. , Dong R. , Chen G. , and Zheng S. , MicroRNA-222 Modulates Liver Fibrosis in a Murine Model of Biliary Atresia, Biochemical and Biophysical Research Communications. (2014) 446, no. 1, 155–159, 10.1016/j.bbrc.2014.02.065, 2-s2.0-84897968739, 24569080. 24569080

[23] Dong R. , Zheng Y. , Chen G. , Zhao R. , Zhou Z. , and Zheng S. , miR‐222 Overexpression May Contribute to Liver Fibrosis in Biliary Atresia by Targeting PPP2R2A, Journal of Pediatric Gastroenterology and Nutrition. (2015) 60, no. 1, 84–90, 10.1097/mpg.0000000000000573, 2-s2.0-84920864222, 25238119. 25238119

[24] Wang R. and Gao Y. , Long Non-Coding RNA Growth Arrest-Specific 5 Inhibits Liver Fibrogenesis in Biliary Atresia by Interacting With MicroRNA-222 and Repressing IGF1/AKT Signaling, Pediatrics. (2023) 12, no. 12, 2107–2120, 10.21037/tp-23-424. PMC10772835 38197105

[25] Ogawa T. , Enomoto M. , Fujii H. , Sekiya Y. , Yoshizato K. , Ikeda K. , and Kawada N. , MicroRNA-221/222 Upregulation Indicates the Activation of Stellate Cells and the Progression of Liver Fibrosis, Gut. (2012) 61, no. 11, 1600–1609, 10.1136/gutjnl-2011-300717, 2-s2.0-84867002762, 22267590. 22267590

[26] Xiao Y. , Zhou Y. , Chen Y. , Zhou K. , Wen J. , Wang Y. , Wang J. , and Cai W. , The Expression of Epithelial-Mesenchymal Transition-Related Proteins in Biliary Epithelial Cells Is Associated With Liver Fibrosis in Biliary Atresia, Pediatric Research. (2015) 77, no. 2, 310–315, 10.1038/pr.2014.181, 2-s2.0-84927129326, 25406900. 25406900

[27] Hu P. , Guo J. , Zhao B. , Zhang Z. , Zhu J. , and Liu F. , CircCHD2/miR-200b-3p/HLF Axis Promotes Liver Cirrhosis, Journal of Environmental Pathology, Toxicology and Oncology. (2022) 41, no. 4, 1–10, 10.1615/JEnvironPatholToxicolOncol.2022041823, 36374958. 36374958

[28] Cheng B. , Zhu Q. , Lin W. , and Wang L. , MicroRNA-122 Inhibits Epithelial-Mesenchymal Transition of Hepatic Stellate Cells Induced by the TGF-*β*1/Smad Signaling Pathway, Experimental and Therapeutic Medicine. (2019) 17, no. 1, 284–290, 10.3892/etm.2018.6962, 30651793. 30651793 PMC6307443

[29] Bala S. , Zhuang Y. , Nagesh P. T. , Catalano D. , Zivny A. , Wang Y. , Xie J. , Gao G. , and Szabo G. , Therapeutic Inhibition of miR-155 Attenuates Liver Fibrosis via STAT3 Signaling, Molecular Therapy Nucleic Acids. (2023) 33, 413–427, 10.1016/j.omtn.2023.07.012. 37547286 PMC10403732

[30] Ariyachet C. , Chuaypen N. , Kaewsapsak P. , Chantaravisoot N. , Jindatip D. , Potikanond S. , and Tangkijvanich P. , MicroRNA-223 Suppresses Human Hepatic Stellate Cell Activation Partly via Regulating the Actin Cytoskeleton and Alleviates Fibrosis in Organoid Models of Liver Injury, International Journal of Molecular Sciences. (2022) 23, no. 16, 10.3390/ijms23169380. PMC9409493 36012644

[31] Lu H. , Zhang R. , Zhang S. , Li Y. , Liu Y. , Xiong Y. , Yu X. , Lan T. , Li X. , Wang M. , Liu Z. , Zhang G. , Li J. , and Chen S. , HSC-Derived Exosomal miR-199a-5p Promotes HSC Activation and Hepatocyte EMT via Targeting SIRT1 in Hepatic Fibrosis, International Immunopharmacology. (2023) 124, Pt. B, 111002, 10.1016/j.intimp.2023.111002, 37804655. 37804655

[32] Li X. , Chen Y. , Wu S. , He J. , Lou L. , Ye W. , and Wang J. , MicroRNA-34a And MicroRNA-34c Promote the Activation of Human Hepatic Stellate Cells by Targeting Peroxisome Proliferator-Activated Receptor *γ* , Molecular Medicine Reports. (2015) 11, no. 2, 1017–1024, 10.3892/mmr.2014.2846, 2-s2.0-84916229794, 25370690. 25370690

[33] Song L. , Chen T. Y. , Zhao X. J. , Xu Q. , Jiao R. Q. , Li J. M. , and Kong L. D. , Pterostilbene Prevents Hepatocyte Epithelial-Mesenchymal Transition in Fructose-Induced Liver Fibrosis Through Suppressing miR‐34a/Sirt1/p53 and TGF-*β*1/Smads Signalling, British Journal of Pharmacology. (2019) 176, no. 11, 1619–1634, 10.1111/bph.14573, 2-s2.0-85065046531, 30632134. 30632134 PMC6514378

[34] Tian X. F. , Ji F. J. , Zang H. L. , and Cao H. , Activation of the miR-34a/SIRT1/p53 Signaling Pathway Contributes to the Progress of Liver Fibrosis via Inducing Apoptosis in Hepatocytes but Not in HSCs, PLoS One. (2016) 11, no. 7, e0158657, 10.1371/journal.pone.0158657, 2-s2.0-84978636812, 27387128. 27387128 PMC4936740

[35] Zhao M. , Qi Q. , Liu S. , Huang R. , Shen J. , Zhu Y. , Chai J. , Zheng H. , Wu H. , Liu H. , and Liu H. , MicroRNA-34a: A Novel Therapeutic Target in Fibrosis, Frontiers in Physiology. (2022) 13, 895242, 10.3389/fphys.2022.895242. 35795649 PMC9250967

[36] Kang H. , Luo J. , Wang C. , Hong Y. , Ye M. , Ding Y. , Zhao Q. , and Chang Y. , miR-192 Inhibits the Activation of Hepatic Stellate Cells by Targeting Rictor, Open Medicine (Wars). (2023) 18, no. 1, 20230879, 10.1515/med-2023-0879. PMC10751890 38152335

[37] Yoneyama T. , Ueno T. , Masahata K. , Toyama C. , Maeda A. , Tazuke Y. , Oue T. , Miyagawa S. , and Okuyama H. , Elevation of microRNA-214 Is Associated With Progression of Liver Fibrosis in Patients With Biliary Atresia, Pediatric Surgery International. (2022) 38, no. 1, 115–122, 10.1007/s00383-021-05009-7, 34546403. 34546403

[38] Ma L. , Yang X. , Wei R. , Ye T. , Zhou J.-K. , Wen M. , Men R. , Li P. , Dong B. , Liu L. , Fu X. , Xu H. , Aqeilan R. I. , Wei Y.-Q. , Yang L. , and Peng Y. , MicroRNA-214 Promotes Hepatic Stellate Cell Activation and Liver Fibrosis by Suppressing Sufu Expression, Cell Death & Disease. (2018) 9, no. 7, 10.1038/s41419-018-0752-1, 2-s2.0-85048825487, 29915227. PMC6006298 29915227

[39] Li H. , Liu T. , Yang Y. , Cho W. C. , Flynn R. J. , Harandi M. F. , Song H. , Luo X. , and Zheng Y. , Interplays of Liver Fibrosis-Associated MicroRNAs: Molecular Mechanisms and Implications in Diagnosis and Therapy, Genes & Diseases. (2023) 10, no. 4, 1457–1469, 10.1016/j.gendis.2022.08.013, 37397560. 37397560 PMC10311052

[40] Tan Z. , Sun H. , Xue T. , Gan C. , Liu H. , Xie Y. , Yao Y. , and Ye T. , Liver Fibrosis: Therapeutic Targets and Advances in Drug Therapy, Frontiers in Cell and Developmental Biology. (2021) 9, 730176, 10.3389/fcell.2021.730176. 34621747 PMC8490799

[41] Xiao Y. , Wang J. , Chen Y. , Zhou K. , Wen J. , Wang Y. , Zhou Y. , Pan W. , and Cai W. , Up-Regulation of miR-200b in Biliary Atresia Patients Accelerates Proliferation and Migration of Hepatic Stallate Cells by Activating PI3K/Akt Signaling, Cellular Signalling. (2014) 26, no. 5, 925–932, 10.1016/j.cellsig.2014.01.003, 2-s2.0-84893523352, 24412919. 24412919

[42] Xia Y. , Luo Q. , Gao Q. , Huang C. , Chen P. , Zou Y. , Chen X. , Liu W. , and Chen Z. , SIRT1 Activation Ameliorates Rhesus Monkey Liver Fibrosis by Inhibiting the TGF-*β*/Smad Signaling Pathway, Chemico-Biological Interactions. (2024) 394, 110979, 10.1016/j.cbi.2024.110979, 38555046. 38555046

[43] Jiao P. , Wang X.-P. , Luoreng Z.-M. , Yang J. , Jia L. , Ma Y. , and Wei D.-W. , miR-223: An Effective Regulator of Immune Cell Differentiation and Inflammation, International Journal of Biological Sciences. (2021) 17, no. 9, 2308–2322, 10.7150/ijbs.59876, 34239357. 34239357 PMC8241730

[44] Yuan S. , Wu Q. , Wang Z. , Che Y. , Zheng S. , Chen Y. , Zhong X. , and Shi F. , miR-223: An Immune Regulator in Infectious Disorders, Frontiers in Immunology. (2021) 12, 781815, 10.3389/fimmu.2021.781815, 34956210. 34956210 PMC8702553

[45] Shaker O. G. and Senousy M. A. , Serum MicroRNAs as Predictors for Liver Fibrosis Staging in Hepatitis C Virus-Associated Chronic Liver Disease Patients, Viral Hepatitis Journal. (2017) 24, no. 8, 636–644, 10.1111/jvh.12696, 2-s2.0-85015155998, 28211229. 28211229

[46] Gunaydin M. and Bozkurter Cil A. T. , Progressive Familial Intrahepatic Cholestasis: Diagnosis, Management, and Treatment, Hepatic Medicine: Evidence and Research. (2018) 2018, 95–104, 10.2147/HMER.S137209.eCollection. PMC6136920 30237746

[47] Kwon Y. , Ahn Y. J. , Yang J. , Kim E. S. , Choe Y. H. , Lee S. , and Kim M. J. , Long-Term Outcomes of Liver Transplantation for Biliary Atresia and Results of Policy Changes: Over 20 Years of Follow-Up Experience, Frontiers in Pediatrics. (2024) 11, 1242009, 10.3389/fped.2023.1242009. 38495838 PMC10940458

[48] Lemoine C. P. , LeShock J. P. , Brandt K. A. , and Superina R. , Primary Liver Transplantation Vs. Transplant After Kasai Portoenterostomy for Infants With Biliary Atresia, Clinical Medicine. (2022) 11, no. 11, 10.3390/jcm11113012, 35683401. PMC9181323 35683401

[49] Kennedy I. , Francis H. , Meng F. , Glaser S. , and Alpini G. , Diagnostic and Therapeutic Potentials of MicroRNAs in Cholangiopathies, Research. (2017) 1, no. 1, 34–41, 10.1016/j.livres.2017.03.003, 2-s2.0-85053346905. PMC5659325 29085701

